# Influence of ancient glacial periods on the Andean fauna: the case of the pampas cat (*Leopardus colocolo*)

**DOI:** 10.1186/1471-2148-9-68

**Published:** 2009-03-30

**Authors:** Daniel Cossíos, Mauro Lucherini, Manuel Ruiz-García, Bernard Angers

**Affiliations:** 1 Département de Sciences Biologiques, Université de Montréal, C.P.: 6128, Succ. Centre-Ville, Montréal, H3C 3J7, Canada; 2 GECM, Cátedra de Fisiología Animal, Departamento de Biología, Bioquímica y Farmacia, Universidad Nacional del Sur-CONICET, San Juan 670, 8000 Bahía Blanca, Argentina; 3 Departamento de Biología, Facultad de Ciencias, Pontificia Universidad Javeriana, Cra 7A No 43-82, Bogotá DC, Colombia

## Abstract

**Background:**

While numerous studies revealed the major role of environmental changes of the Quaternary on the evolution of biodiversity, research on the influence of that period on current South-American fauna is scarce and have usually focused on lowland regions. In this study, the genetic structure of the pampas cat (*Leopardus colocolo*), a widely distributed felid, was determined and linked to ancient climate fluctuations on the Andean region.

**Results:**

Using both mitochondrial sequences and nuclear microsatellites, we inferred the existence of at least four groups of populations in the central Andes, while other three localities, with little sample sizes (n = 3), presented differences in only one of these markers. The distribution of these groups is correlated to latitude, with a central area characterized by admixture of numerous mitochondrial clades. This suggests colonization from at least three glacial refuges and a contact zone between 20 degrees and 23 degrees S following a glaciation event. The similar coalescence times of the mitochondrial haplotypes indicated that the major clades split approximately one million years ago, likely during the Pre-Pastonian glacial period (0.80 – 1.30 MYA), followed by a demographic expansion in every clade during the Aftonian interglacial period (0.45 – 0.62 MYA). Interestingly, this structure roughly corresponds to the current recognised distribution of morphological subspecies.

**Conclusion:**

The four groups of populations identified here must be considered different management units, and we propose the three localities showing differences in only mtDNA or ncDNA as provisional management units. The results revealed the influence of ancient climate fluctuations on the evolutionary history of this species. It is expected that the other species of land vertebrates with a smaller or similar mobility have been affected in the same manner by the glacial and interglacial periods in the central Andes

## Background

Previous bio- and phylogeographic studies in the northern hemisphere revealed the major role of the Quaternary in the evolution of biodiversity. Important climatic fluctuations resulted in alternations of glacial and interglacial periods that profoundly influenced the distribution of habitats, dispersal and isolation among populations [e.g. [1-4]]. In South America, climate fluctuation periods were equivalent to those known for the northern hemisphere [5,6]. During the glacial periods, glacier boundaries descended hundreds of meters, and advanced dramatically in the southern part of the continent [5,7,8]. In the central Andes, although late Quaternary glaciations eliminated the material evidence of glacier advances older than 200 000 years [5], research has shown that the distribution of plant species and habitats cycled with climatic changes, descending to lower elevations during periods of reduced temperature [9,10]. However, studies on the influence of the Quaternary on current South-American biodiversity are scarce and have usually focused on lowland regions [11,12].

Species with broad ranges of distribution can be informative regarding glacial refugia, dispersal pathways and contact zones [13,14] and, then, provide useful insights about the role of climatic changes on biodiversity. The pampas cat (*Leopardus colocolo*) displays a large distribution in South America, from Central Ecuador to Patagonia, in a variety of habitats [15,16]. Based on morphological characteristics, between 8 and 11 subspecies are currently recognized [15,17,18] and the scission of this taxon into three different species was proposed by García-Perea [17]: *L. colocolo *for populations distributed on the western slope of the southern Andes; *L. pajeros *distributed along the Andes; and *L. braccatus *found to the east of the Andes, in Brazil, Uruguay and Paraguay. Previous studies performed on mitochondrial genome [19,20] also revealed that the Andean pampas cat populations are genetically structured and may have experienced significant and lengthy periods of isolation and reduced gene flow.

In spite of its wide distribution, the pampas cat is one of the less known felids [21] and its status is affected by a variety of threats, comprising habitat loss and fragmentation, hunting for traditional reasons and decline of prey populations [22,23]. Lack of evaluation of its conservation status through its range causes the pampas cat to be considered as vulnerable [24] and to be included in the IUCN Near Threatened (NT) category [25].

The aim of the present study was to link the genetic diversity of the pampas cat throughout the central Andes to ancient climate fluctuations. To address this objective, the genetic structure of the pampas cat in 19 localities over a distance of more than 4000 km was inferred with both mitochondrial and nuclear genomes, using non-invasive sampling.

## Results

### Species identification and mtDNA variability

**Figure 1:**
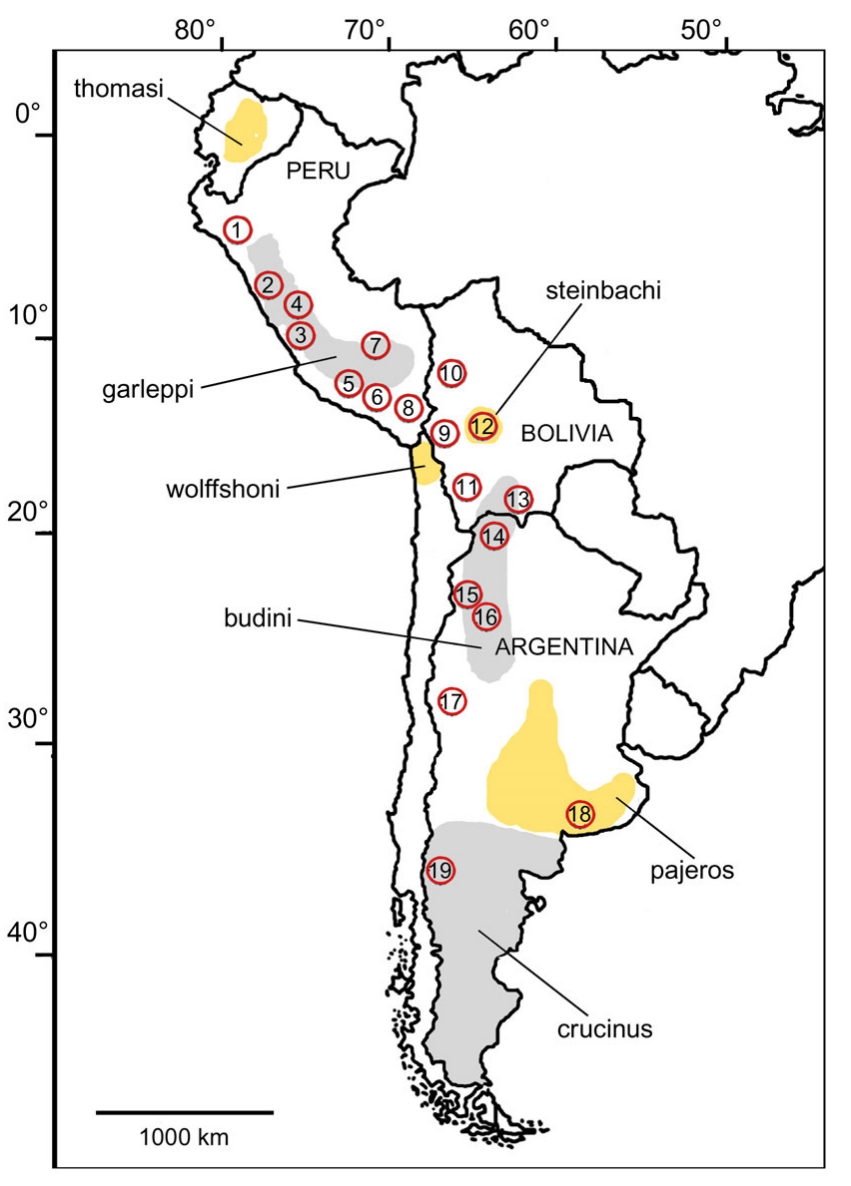
**Pampas cat sampled localities**. The locality numbers correspond to those in Table 1. The grey and yellow areas refer to the approximate distribution of the pampas cat subspecies mentioned in this research, as proposed by García-Perea (1994).

A total of 39 skin samples and 532 faecal samples from 19 localities were analyzed (Figure 1; Table 1). Of the faecal samples, 406 were unambiguously assigned to pampas cats according to the mtDNA *16S *region. The remaining samples were assigned to Andean cats *Leopardus jacobita *(40), domestic cats (11) or canid species (32). A small number of faecal samples (43) failed to be amplified and could not be identified. All skins were assigned to pampas cats.

**Table 1:** Sampled localities, geographical coordinates and sample sizes

	*Coordinates*			
			
*Locality*	S	W	*samples*	*individuals*
1-Lambayeque	06°13'	79°20'	3 (1)	3
2-Ancash	09°33'–10°12'	76°56'–77°23'	38 (1)	19
3-Yauyos/Canchayllo	11°50'–12°07'	75°41'–76°01'	40 (1)	19
4-Junin National Reserve	11°00'	76°07'	27 (2)	12
5-Ayacucho	13°45'–15°00'	73°46'–74°03'	15 (3)	8
6-Arequipa	15°06'–15°41'	70°40'–72°40'	8 (1)	6
7-Cuzco	13°47'	71°14'	1 (1)	1
8-Tacna/Puno	16°36'–17°25'	69°25'–70°03'	19 (19)	19
9-La Paz/Oruro	17°08'–18°26'	68°07'–69°22'	24	12
10-Apolobamba	14°45'	69°00'	2	2
11-Potosi	20°05'–22°09'	67°14'–67°53'	32	15
12-Cochabamba	17°35'	66°24'	3	3
13-Tarija	21°08'	65°04'	2 (2)	2
14-Jujuy	21°57'–23°04'	66°03'–66°18'	58	28
15-Catamarca/Salta	25°13'–26°39'	66°43'–67°39'	35	30
16-Tucuman	26°30'	65°48'	6	5
17-San Juan/Mendoza	29°10'–35°59'	69°21'–69°49'	8	7
18-Buenos Aires	38°10'–38°49'	62°16'–63°15'	5 (5)	5
19-Pilcaniyeu	41°7'	70°40'	3 (3)	3

Total			329 (39)	199

After the total number of pampas cat samples, number of skin samples is indicated in parentheses. Number of individuals as identified from microsatellite data.

**Figure 2:**
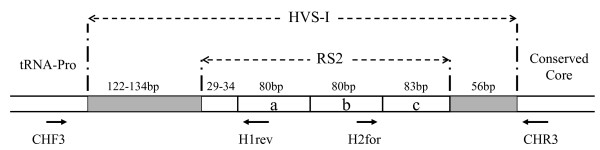
**Pampas cat hypervariable sequence I (HVS-I)**. The position of the primers used in this study is shown. In this example, the repeated sequence 2 (RS2) contains three tandem repeats named here a, b and c. Although the primer H1rev anneals on the a, b and c repetitions and the primer H2for anneals on the a and b repetitions, only their positions on the sequences used in this research are shown.

The length of two mitochondrial control region segments combined varied between 336 and 351 bp. Two variable mononucleotide repeats (T)_3–7 _(C)_3–11 _were identified but not considered for the analyses. Without considering the RS2 region composed of a variable number of large tandem repeats, the pampas cat *HVS-I *region contains between 178 and 190 bp (Figure 2). The range known for other felid species is 231–245 bp [26-28].

**Figure 3:**
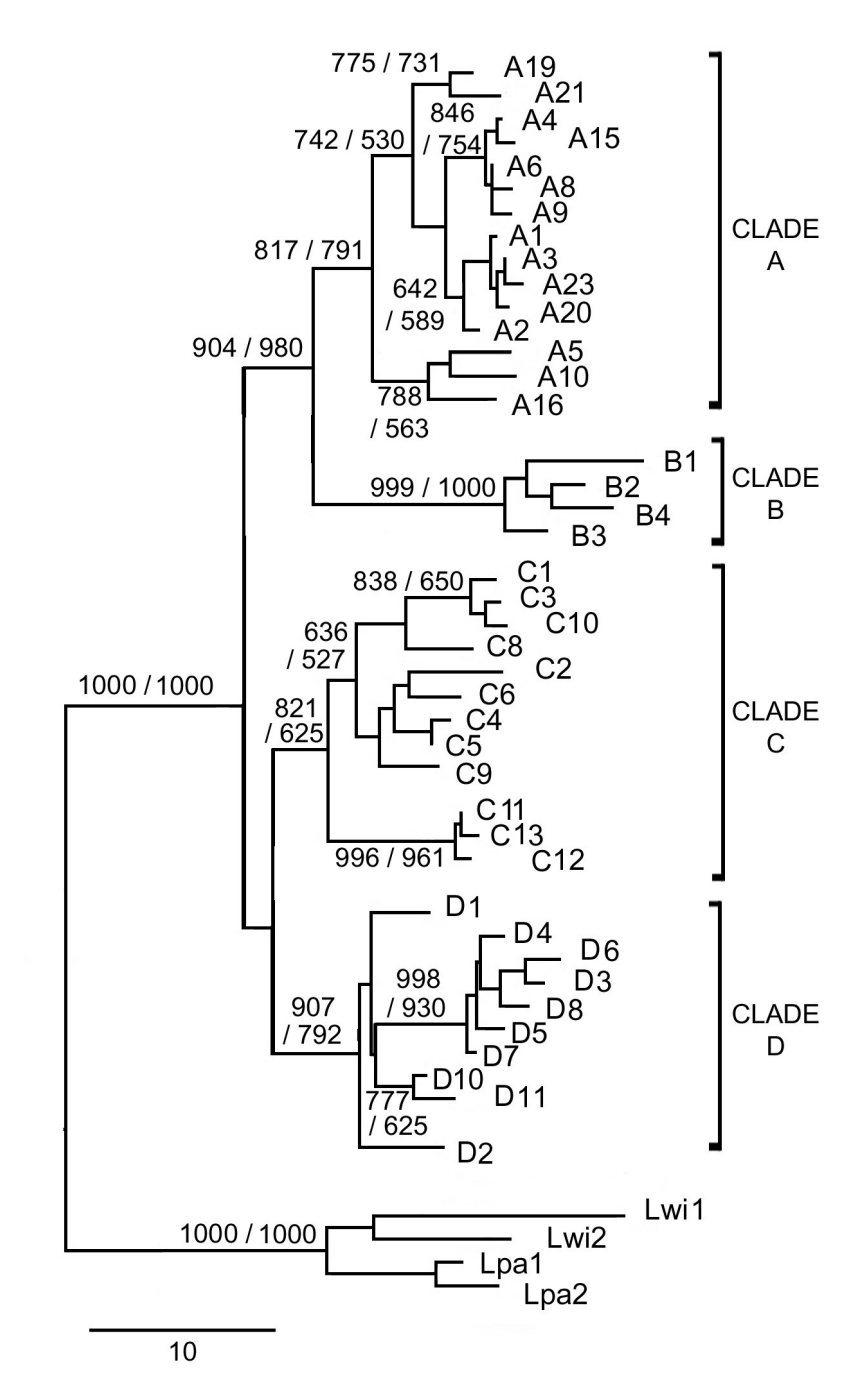
**Neighbour-joining tree of the observed HVS-Isequences**. Labels correspond to haplotype identification numbers. Only bootstrap values over 50% of 1000 bootstraps are shown, corresponding to the NJ/ML analyses. Two ocelots (Lpa1 and Lpa2) and two margays (Lwi1 and Lwi2) were used as outgroups. Scale bar represents an interval of Tamura-Nei genetic distance.

SSCP analyses revealed a single allele per sample, excluding the presence of mtDNA sequences transferred into the nuclear genome (Numts) [28]. The SSCP survey of the 406 samples and the sequencing revealed a total of 41 *HVS-I *haplotypes and 94 variable sites. Phylogenetic relationships inferred among these haplotypes were consistent for both NJ and ML methods. The haplotypes clustered into four major clades, named hereafter A to D, strongly supported by bootstrap values (Figure 3). However, relationships among clades were not completely resolved.

**Figure 4:**
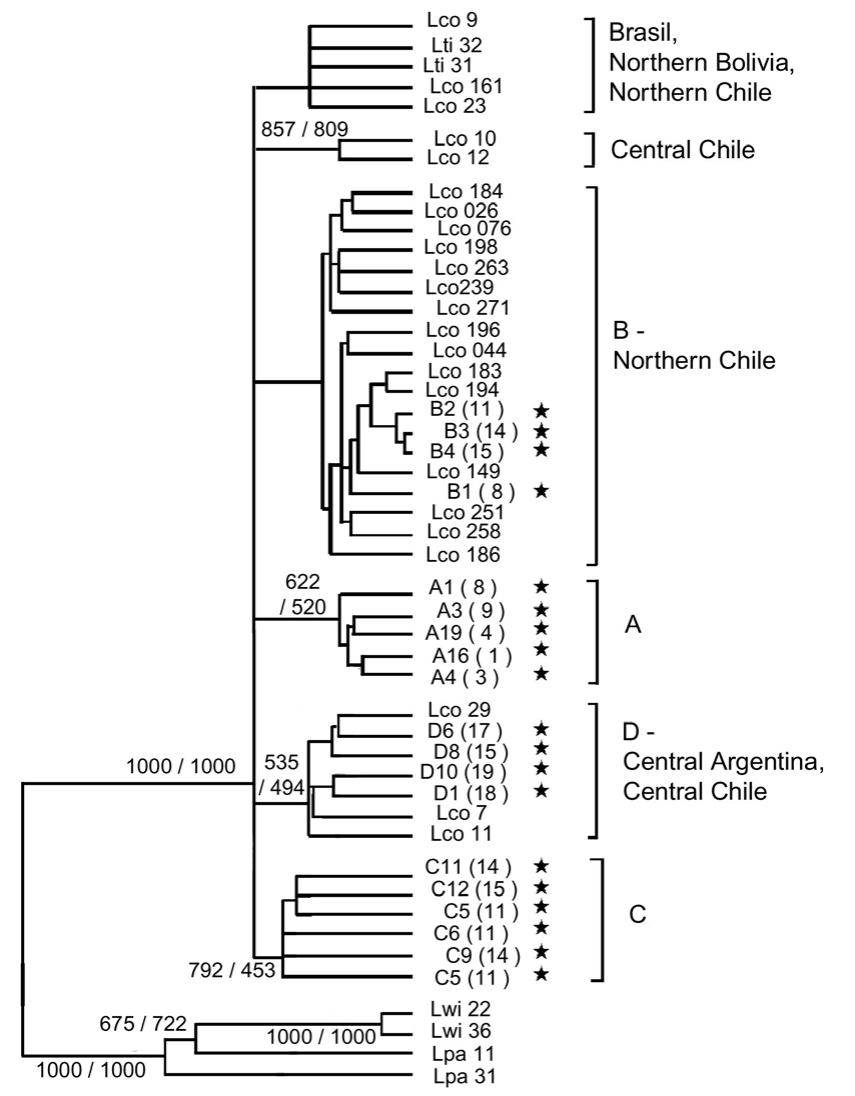
**Neighbour-joining tree of the combined observed NADH-5 – ATP-8 sequences**. Sequences obtained in this study are marked with a star and their labels indicate their haplotype and, between parentheses, their localities. Additional sequences from Chile, Bolivia and Brasil were previously released by Johnson *et al. *(1998) and Napolitano *et al. *(2008), and their labels correspond to individual identification numbers. For each cluster, the correspondent clades of the *HVS-I *are indicated for the individuals sequenced in this research, as well as the geographical origin of the samples analysed by the other authors. Only bootstrap values over 50%of 1000 bootstraps are shown, corresponding to the NJ/ML analyses. Two ocelots (Lpa11 and Lpa31) and two margays (Lwi22 and Lwi36), from Johnson *et al. *(1998), were used as outgroups.

Sequencing of the *NADH-5 *and *ATP-8 *genes revealed 29 sites of additional variation for the 19 individuals selected from our sample. These individuals were selected randomly, with the constraints of choosing individuals with different *HVS-I *haplotypes and covering all the major *HVS-I *clades. The phylogenetic tree inferred using both the *NADH-5 *and *ATP-8 *genes was fully congruent with the one performed with *HVS-I *and allowed the four major clades to be recovered, while the resolution was lower. When individuals from previous studies [20,29] were included into the present dataset, a total of 41 variable sites was detected for *NADH-5 *and *ATP-8 *genes. Most of these additional individuals clustered within clade B (15) and D (3) (Figure 4) while individuals geographically distant from the Andean region, located in central Chile and Brazil, formed two additional clades. Interestingly, individuals presenting *HVS-I *haplotypes of the clades A and C formed two clusters not identified by previous studies.

### Individual identification and population diversity

Microsatellite amplifications provided results for 290 (71%) of the 406 pampas cat faecal samples and for the 39 skin samples (100%) for a total of 329 samples. The microsatellites were highly variable, with 10–21 alleles per locus (Table 2) and the probability of sampling two different individuals with the same genotype ranged between 4.80 × 10^-3 ^and 2.88 × 10^-16^. Unique multilocus genotypes were recorded for 99 faecal samples (30%). However, in several cases, two or more samples displayed the very same multilocus genotype indicating that the same individual had been sampled several times. According to the low probability of obtaining the same genotype in different individuals, samples with the same genotype were assigned to a unique individual, providing a final sample size of 199 pampas cat individuals. The number of individuals per locality varied from 5 to 30, except for 6 localities that had a sample size of 4 individuals or less (Table 1). Localities with sampling size lower than four individuals were not included in the following analyses, unless mentioned in the text.

**Table 2:** Haplotype and microsatellite diversity in pampas cat

							*Microsatellites*										
							
Locality	mtDNA (HVS-I)						Fca24		Fca31		Fca45		Fca96		Fca294		Total
	
	n	H	hd	S	P	π × 100	k	He	k	He	k	He	k	He	k	He	k
1	3	1	0	0	0	0	2	0.73	3	0.60	3	0.73	2	0.33	2	0.60	12
2	19	5	0.59	9	2.64	0.96	6	0.49	5	0.69	9	0.74	6	0.58	9	0.78	35
3	19	6	0.81	9	2.64	0.95	4	0.57	6	0.68	8	0.80	9	0.93	7	0.81	34
4	12	5	0.83	13	3.82	1.46	6	0.64	6	0.75	4	0.70	5	0.59	4	0.70	25
5	8	5	0.86	13	3.82	1.25	4	0.65	8	0.84	5	0.63	5	0.68	6	0.77	28
6	6	5	0.93	9	2.64	1.41	3	0.59	6	0.80	5	0.77	5	0.74	5	0.68	24
7	1	1	0	0	0	0	2	1.00	1	0.00	2	1.00	1	0.00	2	1.00	8
8	19	8	0.91	42	12.3	2.6	4	0.56	6	0.75	6	0.74	7	0.76	8	0.87	31
9	12	8	0.92	24	7.06	2.26	4	0.42	5	0.74	6	0.72	7	0.76	7	0.85	29
10	2	1	0	0	0	0	2	0.50	3	0.84	3	0.84	2	0.67	3	0.84	13
11	15	7	0.88	58	17.06	5.2	5	0.62	7	0.71	3	0.45	5	0.74	5	0.36	25
12	3	2	0.67	11	3.23	2.17	4	0.87	4	0.80	2	0.33	3	0.73	3	0.60	16
13	2	1	0	0	0	0	2	0.50	3	0.84	2	0.50	3	0.84	1	0.00	11
14	28	14	0.9	67	19.7	5.18	7	0.68	7	0.67	8	0.70	8	0.86	7	0.35	37
15	30	14	0.8	66	19.41	4.59	7	0.69	12	0.67	9	0.73	8	0.81	12	0.82	48
16	5	4	0.9	38	11.2	4.76	5	0.89	3	0.61	2	0.25	3	0.55	4	0.79	17
17	7	4	0.81	22	6.47	3.25	4	0.74	6	0.86	4	0.75	6	0.74	5	0.79	25
18	5	2	0.6	8	2.35	1.4	3	0.69	5	0.82	4	0.76	4	0.53	4	0.78	20
19	3	2	0.67	3	0.88	0.59	3	0.73	2	0.33	4	0.87	5	0.93	3	0.73	17
Mean values	14.23	6.69	0.83	29.08	8.55	2.71	4.77	0.63	6.31	0.74	5.61	0.67	6	0.71	6.38	0.72	29.08
Total	199	41	0.94	94	27.65	6.09	10	0.79	21	0.89	15	0.88	14	0.83	14	0.79	74

The locality numbers correspond to those on the Table 1. Mean values were calculated with the localities with more than 4 individuals only. Total values were calculated for the entire sample. n = sample size; H = number of haplotypes; hd = haplotype diversity; S = number of polymorphic sites; P = percentage of variable sites; π = nucleotid diversity; k = number of alleles; H_E _= expected heterozygosity.

The total number of alleles per population ranged from 17 to 48 (Table 2), but was correlated to sampling size (Pearson's r = 0.915, P < 0.0001). The total number of alleles estimated for 5 individuals per population (allelic richness, FSTAT 2.9.3) [30] ranged from 17.16 to 24.85, showing little variation between populations. The expected heterozygosity ranged from 0.35 to 0.93 (Table 2) and was not correlated to sampling size (r = 0.013, P = 0.960). None of the localities displayed deviation from HW expectations, indicating that no more than one population was sampled per locality.

For the mtDNA control region, the number of haplotypes per population ranged from 2 to 14 (Table 2) and was also correlated to sampling size (r = 0.884, P < 0.0001). Haplotype diversity values ranged from 0.60 to 0.93 between the sampled localities, and nucleotide diversity varied by one order of magnitude between 0.0059 and 0.0519.

### Population structure

**Figure 5:**
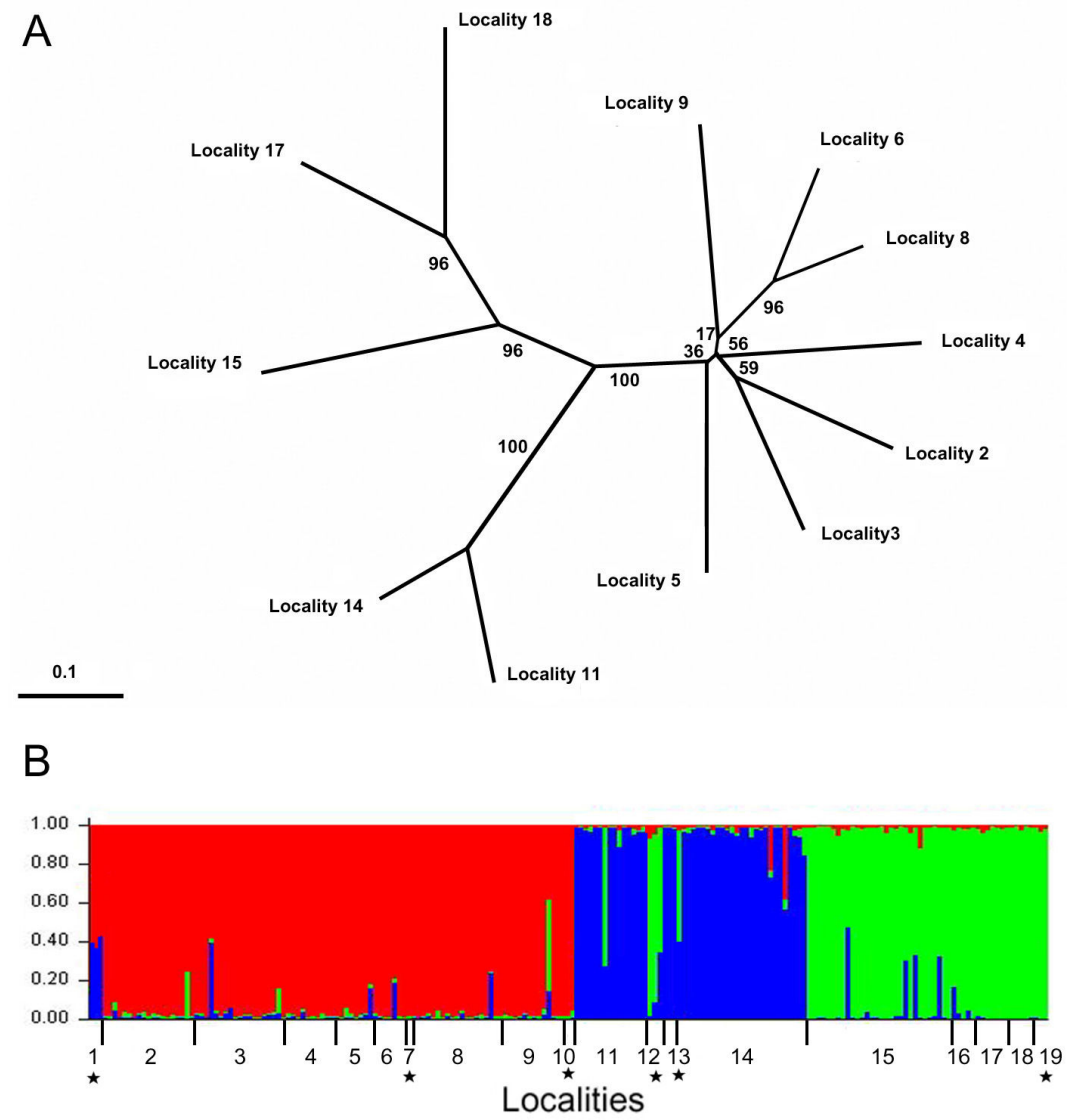
**Population structure inferred from microsatellite data**. A. Neighbour-joining phylogenetic tree of the sampled localities with five or more individuals, constructed using Dce distances and 200 bootstraps on locus. B. Membership coefficients inferred with the program STRUCTURE (Pritchard *et al *2000) for *K *= 3. Each individual is represented by a column and each of the three inferred population groups is represented by a colour. A star indicates localities with less than five individuals, that were not included in the neighbour joining tree showed in (A).

The best value of the ln Pr(X|*K*) obtained with the program STRUCTURE on microsatellite data corresponded to *K *= 3 population groups. These groups included the localities (2–6, 8, 9), (11, 14) and (15–18). Relationships among populations inferred from the microsatellite data supported this grouping, although bootstrap values between populations 2–6, 8 and 9 were low (Figure 5A). AMOVA analysis also supported this structure as the one displaying the highest variation among groups on either mitochondrial or microsatellite data (Table 3).

**Table 3:** AMOVA results for groupings of populations

	% of variation by source		
	
Groupings	Among groups	Among populations/within groups	Within populations
mtDNA			
[2–6, 8, 9][11, 14–18]	58.2	7.16	34.64
[2–6, 8, 9][11, 14][15–18]	62.15	0.22	37.63
[2–6, 8, 9][11, 14, 15][16–18]	57.63	5.44	36.93
Microsatellites			
[2–6, 8, 9][11, 14–18]	8.1	30.24	61.66
[2–6, 8, 9][11, 14][15–18]	43.85	0.93	55.22
[2–6, 8, 9][11, 14, 15][16–18]	18.42	21.86	59.72

Analyses were performed on mtDNA haplotypes using Φ-statistics or microsatellite data using R_ST_-statistics and distances. Localities with less than 5 individuals were not included.

**Figure 6:**
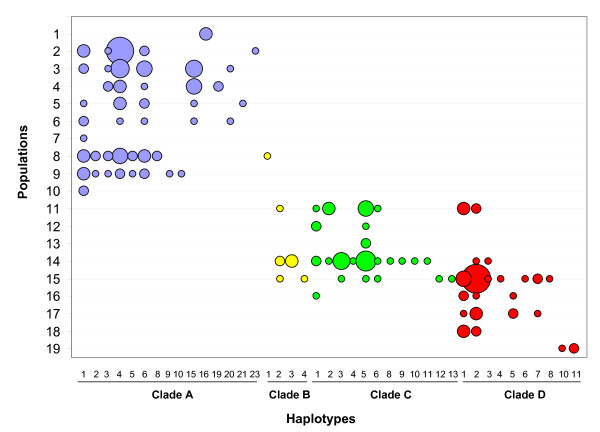
**HVS-I haplotypes distribution and frequencies**. Diameter of circles are proportional to the number of individuals. Numbers assigned to the haplotypes correspond to those on Figure 4.

The distribution of the groups of populations appeared to be clearly correlated to latitude (Figure 6). The first group occupies the area north to 18°S and is formed almost exclusively by individuals of the clade A. The second group, distributed between 20° and 23°S, presents a great proportion of individuals of clade C, although clades B and D are present too. The third group, distributed south to 25°S, contains principally clade D individuals, with some clade B and C individuals in its northern region. The northernmost and the southernmost sampled localities are represented by private haplotypes of the clades A and D, respectively.

Including all localities did not modify the results provided by STRUCTURE: (1–10), (11, 13–14) and (12, 15–19) (Figure 5B) and the population structure remains strongly correlated to geography except for population 12. While geography and mtDNA data suggested including this population in the group (11–13–14), the microsatellite data suggested a closer affiliation with the southernmost group (15–19).

### Evolutionary and demographic history

**Figure 7:**
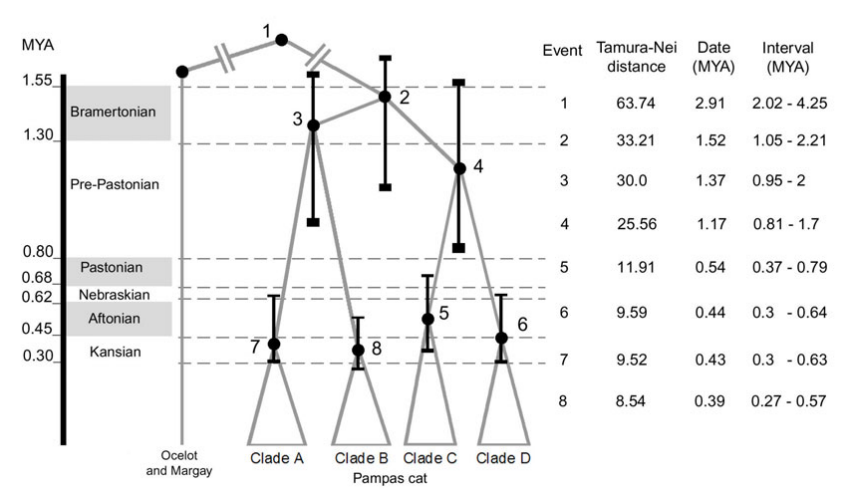
**Divergence times between pampas cat clades and their correspondence with glacial and interglacial periods**. Values were estimated considering a divergence time of 2.91 MY between the pampas cat and the ocelot and the margay, with a confidence interval between 2.02 and 4.25 MY, as estimated by Johnson *et al. *(2006). Interglacial periods are highlighted in grey.

Based on the *HVS-I *sequences, the coalescence times of the clades [A, B, C and D], [A – B], and [C – D] were estimated to 1.52 MY ago (with a confidence interval from 1.05 to 2.21 MYA), 1.37 MY ago (0.95 – 2) and 1.17 (0.81 – 1.7) MY ago respectively. Interestingly, the radiation of the clades A, B, C and D occurred almost simultaneously, with coalescence times of 0.43, 0.39, 0.54 and 0.44 MY ago, respectively. The results for Tamura-Nei distances between clades, coalescence times and confidence intervals are presented in Figure 7.

## Discussion

### Genetic diversity

Previous research has shown that pampas cats present a large genetic diversity at the species level [19]. Results of the present study indicate that a large diversity is also present at populations level and that it is comparable to other non-endangered felid species, like the ocelot and margay [27]. Although we reported a higher *HVS-I *genetic diversity for some pampas cat populations (P = 2.35 – 19.70; Table 2) than for those species (P = 3.71 – 14.73) [27] this can be explained by bigger samples and the presence of highly divergent clades in the most diverse pampas cat populations (Figure 6).

### Genetic structure and geographic distribution

**Figure 8:**
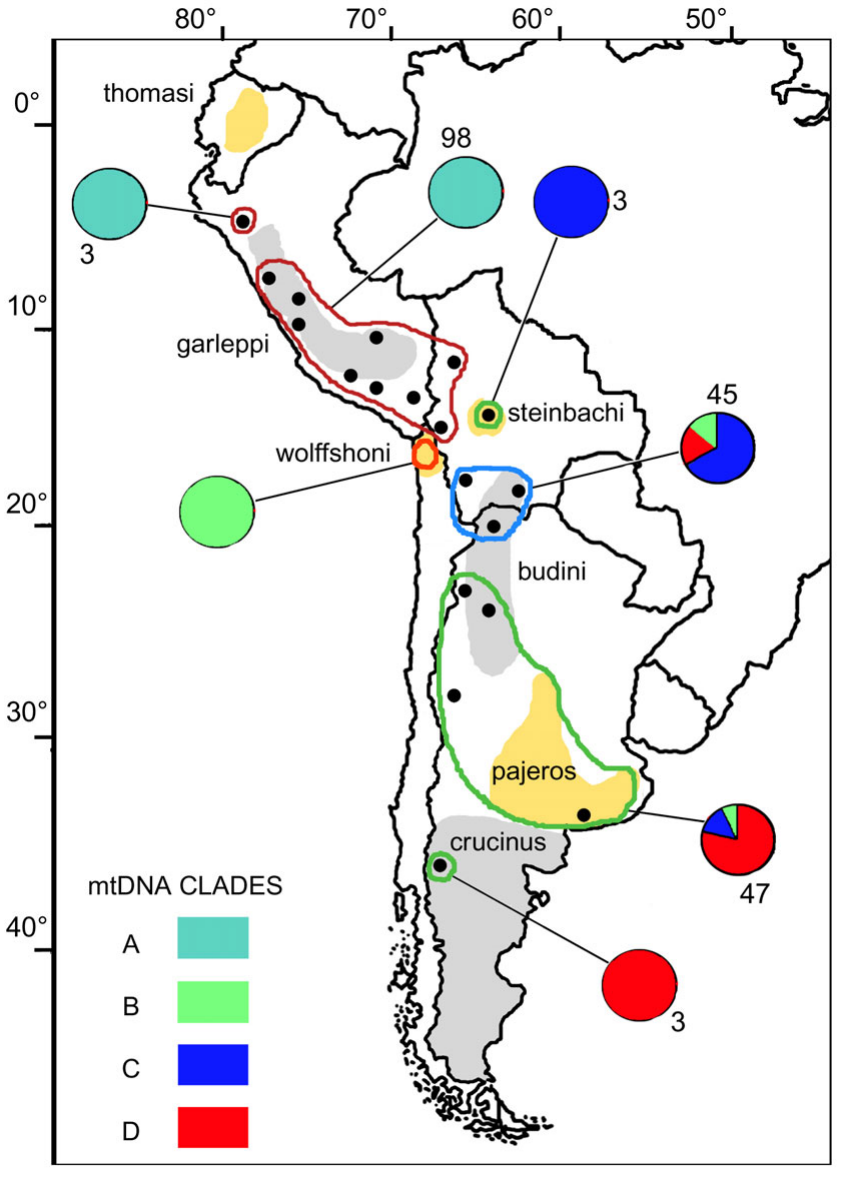
**Distribution of the seven Management Units (MUs) proposed for the pampas cat in the Andean and Argentinean pampas regions, as defined by mtDNA and microsatellite analysis**. The proportion of the mtDNA clades and the sample size are shown for each MU. MUs that are encircled with the same colour presented differences in only mitochondrial or nuclear markers, and those with only 3 individuals sampled are proposed as provisional. The MU without indicated sample size was proposed on the basis of Napolitano *et al *(2008) mtDNA results. The grey and yellow areas refer to the approximate distribution of the pampas cat subspecies mentioned in this research, and their names are indicated. Dots correspond to the sampled localities.

Along the Andean region, analysis of mitochondrial and nuclear genomes revealed the presence of three groups of populations. These groups show a strong geographical structure, with latitudinal separations at 18°–20°S and 23°–25°S. Including results of previous studies based strictly on mitochondrial DNA indicated the existence of a fourth group in northern Chile [20], as well as the two groups out of the Andean region, in Brazil and central Chile [19]. This genetic structure roughly corresponds to the distribution of morphological subspecies described by García Perea [17]. Two of the museum samples, both from locality number 8, were identified as *L. c. garleppi *using the descriptions done by García-Perea [17], although it was not possible to directly assign a genotype to a morphotype for other localities due to the nature of the samples. However, according to the type localities of the described subspecies, the pampas cat groups can be equivalent to *garleppi *(Clade A, 9°–18°S), *budini *(20°–23°S), *pajeros *(25°–38°S) and *wolffshoni *(Clade B northern Chile) subspecies (Figure 8).

The geographical location of populations 12 and 19 coincides with the subspecies *steinbachi *and *crucinus*, respectively, while population 1 is located between the supposed distribution ranges of *thomasi *and *garleppi *(Figure 8). These three localities are characterized by private *HVS-I *haplotypes or ncDNA that differs from that of adjacent populations, and had samples of only 3 individuals. Since a small sample size per locality can affect the results of the STUCTURE program [31] and other analyses, the correspondence between these localities and subspecies needs to be validated by further investigations. The further assignation of these localities to subspecies can be of conservation value since many authors [i.e. [18,19]] do not recognise *steinbachi *and *crucinus *as valid taxa. As this research focused in the central Andean region, further research must be made to describe the genetic structure of the entire species.

The Andean area between 18° and 23°S, approximately, corresponds to an extremely arid belt that separates a northern area, with summer rains, from a southern one, with winter rains [32,33]. This zone is considered to be a barrier between subspecies of other land mammals such as the vicuna *Vicugna vicugna *[34] and the lesser grison *Galictis cuja *[35], and represents a distribution limit for other species, like the long-tailed weasel *Mustela frenata *[36]. In contrast, the genetic structure of the puma *Puma concolor *from the high Andes is not affected by this barrier [37], probably due to a higher dispersal capacity. The pampas cat populations distributed between 18°S and 25°S display three different phenotypes [17] and an important admixture of the clades typical of adjacent areas, suggesting the arid belt as a contact zone.

### Influence of Pleistocene

The topology of the mtDNA trees enabled us to infer two periods of prime importance for the demographic history of the pampas cat. The lack of resolution for the relationships between pampas cat clades (as well as among haplotypes within clades) suggests a rapid radiation.

A first episode comprised the split of the clades A, B, C and D and occurred between the end of the Bramertonian Interglacial (1.30 – 1.55 MYA) and the beginning of the Pre-Pastonian glacial period (0.80 – 1.30 MYA; Figure 7). The Pre-Pastonian corresponds with the most extensive glaciations in southern South America [38]. This period also coincides with the estimated date of the divergence between the major clades of the Andean bird genus *Muscisaxicola *[39], suggesting this period as an important phase in the diversification of the Andean fauna.

The very similar coalescence times of the current haplotypes of the clades A, B, C and D suggest these events took place simultaneously, during a second demographic episode. These splits were likely to be the result of demographic expansions caused by geographically extended phenomena associated with climate change. The calculated mean time for these events overlapped the end of the Kansan glacial period (0.30 – 0.45 MYA) and the Aftonian interglacial (0.45 – 0.62 MYA), the interglacial period being the more likely moment of occurrence for these demographic events (Figure 7).

Interestingly, the divergence time between clades A, B, C and D is similar or longer than between some felid species, such as the Iberian lynx *Lynx pardinus*, Canadian lynx *Lynx canadensis *and Eurasian lynx *Lynx lynx *(between 1.18 and 1.61 MY), kodkod *Leopardus guigna*, little spotted cat *Leopardus tigrinus *andGeoffroy's cat *Leopardus geoffroyi *(0.74–0.93 MY) and ocelot and margay (1.58 MY) [40].

High diversity within clades, times of divergence and monophyletic origin in most of the regions indicated long-term isolation into distinct refuges (glacial)/regions (interglacial) rather than dispersal from a unique refuge. No Pleistocene refugia were previously identified in the central Andes for medium-sized mammals. However, considering the current distribution of the clades and the zones apparently less affected by the glaciations [5], clade A refuge probably existed in central Peru, while clade B and clade D refuges were possibly located in northern Chile and eastern Argentina, respectively. Clade C refuge was probably placed in eastern Bolivia. Individuals with haplotypes of clades B, C and D would subsequently migrate to a contact zone between 20 and 23°S during an interglacial period.

### Implications for conservation

The concepts of "evolutionarily significant units" (ESUs) [41] and "management units" (MUs) [42] were created with the objective of targeting operational units for conservation below the species level. Although the use of the ESUs concept and its importance for prioritising conservation efforts is currently well accepted, its definition was strongly debated [43]. Many authors consider reciprocal monophyly as mandatory for the recognition of ESUs, as proposed by Moritz [42], or subspecies, while for other definitions a different evolutionary history between populations is sufficient [41,44-46]. MUs, in the other hand, are defined as population units with statistically significant differences of allelic frequencies at nuclear or mitochondrial level [42].

The long divergence time among clades, as well as the differentiation at the ncDNA level, highlight the importance of recognizing the four pampas cat population groups identified here (localities 2–10/11, 13–14/15–18/northern Chile) as different units for conservation in the Andean region. Since they have different allelic frequencies at both, mtDNA and ncDNA levels, all of these groups must to be recognised as MUs. In addition, we recommend the recognition of the localities 1, 12 and 19 as provisional MUs, since only private haplotypes were found for them (localities 1 and 19) or they differ at ncDNA level from adjacent MUs (locality 12; Figure 8).

The existence of an admixture zone in the central Andean region results in a lack of reciprocal monophyly between all the four population groups and makes their recognition as subspecies or ESUs controversial. However, cases of hybridization between subspecies or species are common in nature [47], of particular interest being the identification of hybridization areas for tigrinas, Geoffroy's cats and pampas cats [19,48]. In this sense, pampas cats from central Andes can be regarded as a complex of at least four ESUs/subspecies, one of them being the result of a past hybridization event.

## Conclusion

Through the analyses of both mitochondrial and nuclear DNA, we inferred the population structure of the pampas cat in a broad portion of the Andean region, showing the existence of at least four groups of populations that must be considered different management units, and three other localities proposed here as provisional management units. The results revealed the influence of ancient climate fluctuations on the evolutionary history of this species, suggesting the split of four mtDNA clades during the Pre-Pastonian glacial period, long term isolation and further population expansions during the Aftonian interglacial. The pampas cat is a species with a relatively high mobility. It is expected that the other species of land vertebrates with a smaller or similar mobility have been affected in the same manner by the glacial and interglacial periods in the central Andes and, hence, they show similar population structures at present, that should be considered for the future study and conservation of the Andean fauna.

## Methods

### Study localities and sampling

A total of 39 skin samples and 532 faecal samples from 19 localities were analysed (Figure 1; Table 1). Samples were collected in the Andes of Argentina, Bolivia and Peru, covering more than 4350 km from 06°13'S to 41°07'S, with the exception of 5 tissue samples collected in the Argentine Pampas lowland of Buenos Aires province. Faecal samples were collected opportunistically in the field and kept in paper bags surrounded by silica gel in excess or in vials with ethanol for their transportation to the laboratory [49]. Once in the laboratory, samples were kept at -20°C, without silica gel, until DNA extraction. Skin samples were taken from stuffed animals owned by villagers or from museum specimens. For skin samples, approximately 0.5–1 cm^2 ^was cut from the ear or, if that was not possible, from other areas, and kept in individual paper bags, in dry and cool conditions [50].

### DNA extraction

DNA from skin samples was isolated by the standard method of proteinase K digestion, phenol-chloroform extraction and precipitation with ethanol [51]. DNA from faeces was isolated with the QIAamp^® ^DNA Stool Mini Kit (QIAGEN, Ontario, Canada) according to the manufacturer's instructions, with the following two modifications: i) instead of 180–220 mg, from 200 to 500 mg of the superficial faecal material was used per sample, and ii) the ASL buffer was heated to 70°C before being added to the samples. To prevent and monitor the contamination of the samples during the laboratory processes, pre-PCR and post-PCR activities were carried out in different laboratories and negative controls were included in each batch of extraction and amplification [52,53].

### Species identification

Faecal samples were identified to the species level by a PCR-RFLP method [54]. Briefly, a segment of 257–263 bp of the *16S *mitochondrial gene was amplified by PCR and the product was exposed to the action of several restriction enzymes, resulting in species-specific fragment profiles which can be visualised on agarose gel.

### MtDNA

The hypervariable domain 1 (*HVS-I*) of the mtDNA control region was selected because it is a non-coding sequence that is expected to display high polymorphism within felid species [27,55-57]. Because DNA from faeces and ancient tissues is often degraded [58,59] and prevents the amplification of the complete *HVS-I*, primers were designed to amplify two short segments on the *HVS-I*. In order to design these primers, the complete *HVS-I *region was amplified using fresh tissues from four Peruvian pampas cats with the primers CH3F and CH3R developed by Freeman *et al. *[56]. Both strands were sequenced with a CEQ 8000XL DNA Analysis System (Beckman Coulter Inc., Fullerton, Calif.). These sequences were aligned with the *HVS-I *region of domestic cat (Gene Bank accession number NC_001700), cheetah *Acinonyx jubatus *(NC_00512), margay *Leopardus wiedii *(AF129663S1-2) and ocelot *Leopardus pardalis *(AF129645S1-2) using the program Clustal W [60]. Finally, sequences conserved among species were used to design the felid-specific primers H1rev (5'-CCTGTACATGCTTAATATTC-3') and H2for (5'-ACATAYTATGTATATCGTGC-3') which provide PCR products smaller than 300 bp when used with CH3F and CH3R primers, respectively (Figure 2).

Amplification reactions were carried out in a volume of 12.5 μl containing a final concentration of 20 mM Tris-HCl (pH 8.4), 50 mM KCl, 1.5 mM MgCl_2_, 0.1 mM of each dNTP and 0.8 pM of each primer, 0.8 mg/ml of BSA, 0.2 unit of Taq DNA polymerase, and approximately 20 ng of template DNA. PCR conditions included an initial denaturing step at 92°C for 2 min, 45 cycles of 92°C for 15 s, 52°C for 15 s, and 68°C for 30 s, and a final extension step at 68°C for 5 min. Mitochondrial DNA variation was detected using single-strand conformation polymorphism (SSCP) [61]. The amplified products were electrophoresed on a 6% nondenaturing gel for 11 h 30' at 20 W in 0.5× TBE [62] and visualized using silver nitrate staining [63]. A band was sliced from the gel for each of the different observed conformers and placed in a volume of 35 μl of HPLC-grade water overnight. 2 μl of the dissolved DNA were used for amplification, and the products were sequenced. Because H1rev and H2for primers fit on a sequence of repeated tandems, called RS2 [28] (Figure 2), the product amplified with the CH3F-H1rev pair of primers was sequenced only in the forward direction, while that amplified with the H2for-CH3R primers was sequenced only in reverse. When possible, at least two individuals from different populations were sequenced for each observed conformer, to confirm the reliability of the SSCP protocol.

The mitochondrial genes *NADH-5 *(318 bp) and *ATP-8 *(191 bp) were sequenced for a sub-sample of 19 individuals, using the primers developed by Johnson *et al. *[29], to compare the phylogenetic relationships of the sampled individuals with pampas cats from other regions [20,29]. *HVS-I*, *NADH-5 *and *ATP-8 *sequences have been deposited in GeneBank (accession numbers FJ648644–FJ648684, FJ664428–FJ664446 and FJ664409–FJ664427, respectively).

### Microsatellites

Five microsatellite loci isolated from domestic cats (Fca24, Fca31, Fca45, Fca96 and Fca294) [64] were amplified and screened on acrylamide gels. Amplification reactions were carried out for all the pampas cat samples, with the same PCR conditions indicated above for the mtDNA. For the faecal samples, amplification and screening was made three times, following the multiple tube approach [65] and only the samples showing concordant results were used for data analysis.

### Data analysis

Measures of population genetic diversity, including nucleotide diversity (π) and haplotype diversity (hd) [66] were estimated from mtDNA sequences using the computer program ARLEQUIN 2.00 [67]. Exact tests of population differentiation [68] with a 10000 Markov chain, and estimations of F_ST _between pairs of localities [69] were performed with the same software.

The phylogenetic relationships of the aligned sequences were analysed by distance (neighbour joining, NJ) [70] and maximum likelihood (ML) methods using the program PAUP 4.01b [71]. Gaps were treated as single haplotype variants. After an election using MODELTEST 3.7 [72], the Tamura-Nei model [73] was used for NJ and ML analyses, assuming a gamma distribution for substitution rates across sites (AIC = 2534.70283). Node support was assessed using 1000 bootstrap replicates. Sequences of the equivalent control region segment from two ocelots and two margays were used as outgroups. To assess the extent of differentiation among regions and sampled localities, we performed an analysis of molecular variance (AMOVA) [74], with Φ-statistics, using ARLEQUIN 2.00 [67], with statistical significance tested using 10000 permutations. Several groupings were tested with AMOVA, and the grouping that maximized differentiation among regions and minimized differentiation among localities within regions was assumed to represent to the most parsimonious geographical subdivisions.

To estimate the divergence times between different groups, we considered the *HVS-I *region, a divergence time of 2.91 MY between the pampas cat and the ocelot and the margay, with a confidence interval between 2.02 and 4.25 MY [40], and the mean Tamura-Nei molecular distances between haplotypes of the identified clades as calculated in ARLEQUIN 2.00. All the identified haplotypes were used for each clade. Regions corresponding to deletions or insertions in different species were not considered for this analysis.

For each microsatellite genotype, the probability that two samples with the same genotype represent two different individuals was calculated from the allele frequencies in each sampled locality [75]. To obtain the value for the complete genotype, probabilities for each locus were multiplied, assuming independence of loci, as supported by the linkage map of microsatellite loci in the domestic cat [64].

Measures of expected heterozygosity (H_E_) and number of alleles (k) were estimated using the computer program ARLEQUIN 2.00 [67]. Hardy-Weinberg equilibrium was tested for each sampled locality with the method developed by Guo & Thompson [76] using GENEPOP 3.4 [77]. The population structure was examined for the microsatellite data by a Bayesian clustering method, using STRUCTURE 2.2 [78], with a 100000 burn-in period and 50000 MCMC repetitions, to infer the number of populations (*K*) and to assign individuals to inferred population clusters. Additionally, a neighbour-joining phylogenetic tree of the sampled localities with five or more individuals was constructed with the program POPULATIONS 1.2.28 [79] with the microsatellite data, using Dce distances [80] and 200 bootstraps on locus. As for the mtDNA data, AMOVA was performed on several groupings to test the population structure.

## Authors' contributions

DC conceived the study and participated in its design, carried out the molecular genetic studies and the statistical analysis, and drafted the manuscript. BA coordinated the study, participated in the design and in the statistical analysis, and helped to draft the manuscript. ML and MRG contributed in data acquisition and interpretation, and helped to draft the manuscript. All authors read and approved the final manuscript.
